# Incidental Prophylactic Appendectomy Is Associated with a Profound Microbial Dysbiosis in the Long-Term

**DOI:** 10.3390/microorganisms8040609

**Published:** 2020-04-23

**Authors:** Lidia Sánchez-Alcoholado, José Carlos Fernández-García, Carolina Gutiérrez-Repiso, M Rosa Bernal-López, Luis Ocaña-Wilhelmi, Eduardo García-Fuentes, Isabel Moreno-Indias, Francisco J. Tinahones

**Affiliations:** 1 Unidad de Gestión Clínica de Endocrinología y Nutrición del Hospital Virgen de la Victoria, Instituto de Investigación Biomédica de Málaga (IBIMA), Universidad de Málaga, 29010 Málaga, Spain; l.s.alcoholado@gmail.com (L.S.-A.); josecarlosfdezgarcia@hotmail.com (J.C.F.-G.); gutierrezrepiso@gmail.com (C.G.-R.); 2 Centro de Investigación Biomédica en Red de Fisiopatología de la Obesidad y la Nutrición, CIBERobn, 28029 Madrid, Spain; robelopajiju@yahoo.es (M.R.B.-L.); edugf1@gmail.com (E.G.-F.); 3 Servicio de Medicina Interna, Hospital Regional Universitario de Málaga, Instituto de Investigación Biomédica de Málaga (IBIMA), Universidad de Málaga (UMA), 29010 Málaga, Spain; 4 Department of Surgery, Institute of Biomedical Research of Malaga (IBIMA), Virgen de la Victoria Clinical University Hospital, 29010 Malaga, Spain; luisowilhelmi@hotmail.com; 5 Unidad de Gestión Clínica de Aparato Digestivo, Instituto de Investigación Biomédica de Málaga (IBIMA), Hospital Universitario Virgen de la Victoria, 29010 Málaga, Spain

**Keywords:** incidental prophylactic appendectomy, gut microbiota, appendix, dysbiosis

## Abstract

Incidental prophylactic surgeries are performed in certain situations. Incidental prophylactic appendectomies were common practice within opened bariatric surgeries. The gut microbiota has emerged as an important actor within the homeostasis of the host. A new hypothesis has been formulated about the appendix function in relation to gut microbiota. Our objective was to study the gut microbiota profiles of patients that had suffered from an incidental prophylactic appendectomy during their bariatric surgeries, while comparing them to patients whose appendixes had remained intact. A case-control observational prospective study of 40 patients who underwent bariatric surgery, with or without an incidental prophylactic appendectomy, during 2004–2008 with an evaluation of their gut microbiota populations at the end of 2016 was conducted by sequencing the 16 S rRNA gene by Next Generation Sequencing of patients’ stools and appendix tissues. Patients with their appendix removed showed lower levels of richness and diversity of their gut microbiota populations. *Odoribacter*, *Bilophila*, *Butyricimonas*, and *Faecalibacterium* levels were increased in the Intact group, while *Lachnobacterium* suffered an expansion in the group without the appendix. Moreover, a linear regression *model* introduced the concept that *Butyricimonas* and *Odoribacter* may be implicated in insulin regulation. Thus, gut microbiota should be considered in the decisions of practical surgery, regarding the appendix as a mediator of homeostasis in the host. *Butyricimonas* and *Odoribacter* require further investigation as key bacteria implicated in insulin regulation.

## 1. Introduction

Prophylactic surgeries are performed under a risk-benefit assessment. Incidental prophylactic procedures should be easily performed, inexpensive and should not cause increased morbidity or mortality. However, the repercussions of incidental prophylactic surgeries beyond these premises have not been greatly considered. This is the case for homeostatic repercussions.

Incidental prophylactic appendectomy, which is the process by which the appendix is removed during any other abdominal operation to eliminate the potential for future appendicitis, is a practice still performed in some situations [1]. The fear of appendicitis complications is a consequence for most emergency surgical operations [2]. Ancient evolutionary perspectives considered the vermiform appendix as a rudimentary part of the human intestine in a vestigial form [3]. However, new theories have been recently proposed. The “safe house” hypothesis, which states that the human appendix is a reservoir of healthy microbiota to be replaced in the case of necessity, is one of the most interesting approaches [4].

Gut microbiota has been recognized as an important actor within the metabolic homeostasis of the host [5]. Amongst its functions, it has essential immunological, metabolic, structural and neurological actions for the homeostasis of the host [6]. These new theories regarding gut microbiota and the appendix microbiota should be further studied.

In this study, we aimed to analyze gut microbiota profiles between patients that underwent an incidental prophylactic appendectomy during their bariatric surgeries, in comparison with patients whose appendixes were not removed.

## 2. Materials and Methods

### 2.1. Patients

This was a case-control observational prospective study carried out at the Endocrinology Department of Virgen de la Victoria University Hospital (Málaga, Spain), that included 40 patients who underwent bariatric surgery from 2004 to 2008. All the subjects were of Spanish nationality and lived in the same urban area of Málaga (Spain), with a similar socio-economic status. Patients had a similar diet, because of traditional habits as well as facultative recommendations, both before and after surgery. Out of these 40 patients, 20 patients had had their appendix removed from an incidental prophylactic appendectomy (w/oApp group), and 20 patients had a preserved appendix (Intact group). Patients from both groups were matched based on: age at the time of the bariatric surgery operation, body mass index (BMI), and the metabolic outcomes of their routine (glucose, insulin, triglycerides, cholesterol, HDL-cholesterol, and blood pressure) (see Table 1). Patients underwent bariatric surgery for any of these procedures: Scopinaro (BPD), Gastric bypass (GB) or sleeve gastrectomy (SG), with a balanced number from each group as much as possible. Moreover, the exclusion criteria of the study included the consumption of any microbiota-perturbing products, such as antibiotherapy or probiotics, in the 3 months prior to the fecal sample collection. In addition, records on drug consumption during the post-surgery period were also considered for recruitment. The explanatory scheme of the study design is shown in Supplementary Figure S1. This study was reviewed and approved by the Ethics and Research Committee of the province of Málaga (CEI Provincial de Málaga), Málaga, Spain, (approved on 28/12/2015 within the project “Modificaciones epigenéticas y microbiota en la genesis de la disfunción del tejido adiposo y la Resistencia a la insulina”) and was conducted according to the principles of the Declaration of Helsinki. The participants (who were all volunteers) provided signed consent after being fully informed of the study goal and its characteristics.

### 2.2. Analysis of Anthropometric and Biochemical Variables

Body weight, height, and waist circumference were measured according to standardized procedures. The BMI was calculated as weight (kilograms) divided by height (meters) squared. Blood samples were collected after an overnight fast. Serum levels of glucose, cholesterol, triglycerides and HDL cholesterol were analyzed using a Dimension autoanalyzer (Dade Behring Inc., Deerfield, IL, USA) by enzymatic methods (Randox Laboratories Ltd., Crumlin, UK). The homeostasis model assessment of insulin resistance (HOMA-IR) index was calculated as follows: HOMA-IR = fasting plasma insulin (µU/mL) x fasting plasma glucose (mmol/L)/22.5.

### 2.3. DNA and RNA Extraction

Fecal samples were collected and immediately stored at −80 °C until the analysis. DNA extraction from 200 mg of stool was conducted using the QIAamp DNA stool Mini kit (Qiagen, Hilden, Germany), according to the manufacturer’s instructions. Total RNA was extracted from the fecal appendix samples using the commercial TriPure Isolation Reagent kit (Roche) and treated with DNase (Qiagen, Hilden, Germany). The DNA and RNA concentrations were determined by absorbance at 260 nm (A260). Purity was estimated by determining the A260/A280 ratio. Both measurements were performed with a Nanodrop spectrophotometer (Nanodrop Technologies, Thermofisher, Gibraltar, UK). cDNA was synthesized using SuperScript II reverse transcriptase and random hexamer primers, following the manufacturer’s protocol (Invitrogen, Carlsbad, CA, USA) for RNA.

### 2.4. Microbiota Analysis

Ribosomal 16S rRNA gene sequences were amplified from cDNA using the 16S Metagenomics Kit (Thermo Fisher Scientific, Monza, Italy). The kit included two primer sets that selectively amplify the corresponding hypervariable regions of the 16S region in bacteria: primer set V2–4–8 and primer set V3–6, 7–9. Libraries were created using the Ion Plus Fragment Library Kit (Thermo Fisher Scientific, Monza, Italy). Barcodes were added to each sample using the Ion Xpress Barcode Adapters kit (Thermo Fisher Scientific, Monza, Italy). Emulsion PCR and sequencing of the amplicon libraries were performed on an Ion 520 chip (Ion 520TM Chip Kit), using the Ion Torrent S5TM system and the Ion 520TM/530TM Kit-Chef (Thermo Fisher Scientific, Monza, Italy) according to the manufacturer’s instructions. After sequencing, the individual sequence reads were filtered using Ion Reporter Software V4.0, to remove low quality and polyclonal sequences.

### 2.5. Bioinformatic Analysis

Sequences were further translated into amplicon sequence variants (ASVs) using DADA2 with adapted parameters for Ion Torrent data [7] within the microbiome analysis package QIIME2 (www.qiime2.org) [8]. This was also used for diversity analysis and subsequent taxonomic analysis, through clustering with vsearch [9] and the reference base Greengenes version 13_8 at 99% of identity. The web-tool MicrobiomeAnalyst (www.microbiomeanalyst.ca) [10] was used for further differential abundance analysis through edgeR [11], and a linear discriminant analysis effect size (LEFSE) for searching biomarkers [12]. Venn diagram analysis was performed with Venny 2.1.0 [13].

### 2.6. Statistical Analysis

Biochemical and anthropometrical data were analyzed by the statistical software package SPSS version 22.0 (SPSS Inc., Chicago, IL, USA). Comparisons between pre and post-surgery outcomes within the groups were made with paired tests. Comparisons between the results of both groups of patients were made with the Mann–Whitney test and a Bonferroni post hoc test. Microbiota results were corrected by multiple comparisons, by the Benjamini–Hochberg False Discovery Rate (FDR) correction (*q*-value). Values were considered to be statistically significant when *p* <0.05 or *q*-value < 0.05.

## 3. Results

Table 1 depicts the anthropometric and biochemical variables of the study participants. No significant statistical differences between the groups were found in any studied parameter at the preSurgery or studyTime. However, while patients from both groups experienced significant improvements in parameters such as glucose, cholesterol or HDL-cholesterol, Intact patients did not significantly reduce their triglycerides levels, and w/oApp patients did not significantly improve their insulin levels.

### 3.1. Intact and Appendectomized Patients Differed in Their Gut Microbiota Populations

**Figure 1:**
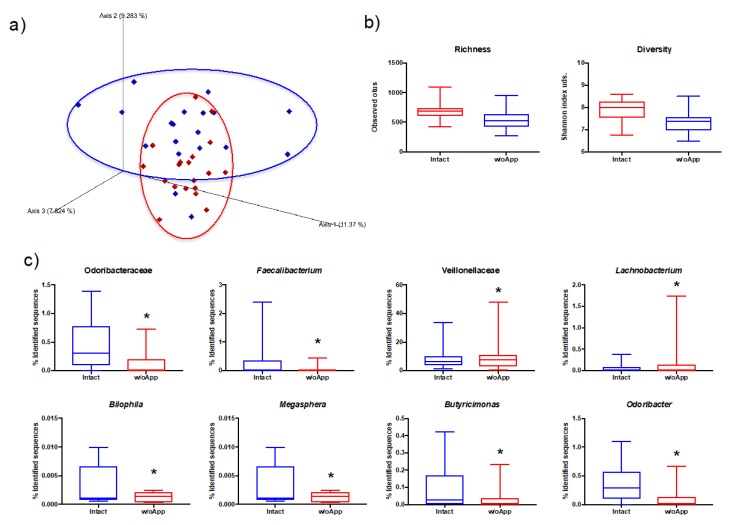
(**a**) Principal Coordinates Analysis (PCoA) of bacterial communities from patients who underwent incidental prophylactic appendectomy and in stool samples from these patients (w/oApp, blue) and from patients with their appendixes intact (Intact, red) during bariatric surgery. (**b**) Estimate richness and diversity indices between stool bacterial communities from Intact and w/oApp patients. (**c**) Differential abundant bacteria between the stool samples from Intact and w/oApp patients analyzed with the method of RNAseq with EdgeR analysis (* indicates FDR-corrected *p*-value < 0.05).

The unweighted UniFrac distance was used to assess the similarity of the samples, revealing statistically significant differences (PERMANOVA, *p* = 0.011) between the microbiota populations of intact patients and those with their appendix removed (Figure 1a) at studyTime. This trend was followed by the diversity analysis, assessed by the richness (observed ASVs index) and diversity (Shannon index), which revealed significant differences in feces between study groups (Figure 1b). Patients from the w/oApp group showed lower richness and diversity of gut microbiota populations than their Intact counterparts.

Differential abundance analysis (Figure 1c) revealed particular differences between groups in fecal samples at the family level: Odoribacteriaceae (*q*-value = 0.011) and Veillonellaceae (*q*-value = 0.048), while at the genus level: Megasphaera (*q*-value = 8.20 × 10^−9^), Odoribacter (*q*-value = 0.003), Bilophila (*q*-value = 0.003), Faecalibacterium (*q*-value = 0.013), Butyricimonas (*q*-value = 0.019) and Lachnobacterium (*q*-value = 0.044). Moreover, a linear discriminant analysis effect size (LEFSE) was used in order to search for biomarkers, finding that Odoribacter (LDAscore = −4.23, *q*-value = 0.015) and Bilophila (LDAscore = −4.54, *q*-value = 0.026) could be representative of the w/oApp group.

### 3.2. Appendix and Stool Microbiota Populations

**Figure 2:**
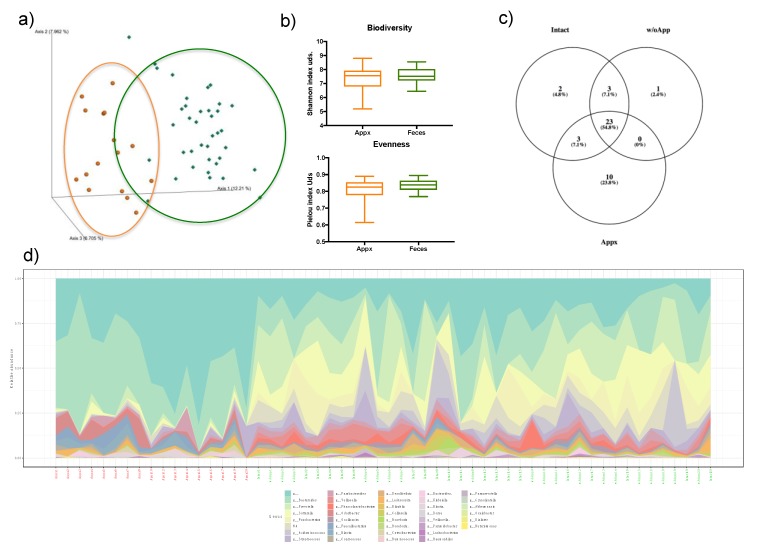
(**a**) Clustering of bacterial communities of the appendix (orange dots) or fecal samples (green diamonds) by principal coordinate analysis (PCoA) using the weighted UniFrac distances. (**b**) Alpha diversity indexes of appendix and fecal samples. (**c**) Venn diagram of the microbiota profiles at family level of the analyzed fecal samples of the w/oApp and Intact groups as well as the appendix tissues. (**d**) Microbiota profiles at genus level of the appendix and fecal samples.

The appendix and stool differed in their microbiota populations, especially in their abundances (Figure 2a, PERMANOVA, weighted Unifraq distances, *p* = 0.001). However, no differences were observed in any measured index of alpha-diversity (Figure 2b). In order to deeply understand these changes and relate them to the Intact and w/oApp populations, an analysis of similarity was performed through Venn diagrams. At the phylum level, six different phyla were shared by appendix and stool samples (Actinobacteria, Bacteroidetes, Firmicutes, Fusobacteria, Proteobacteria, and Sinergistetes), while Lentisphaerae and Verrucomicrobia were only found in Intact and w/oApp stools. At the family level (Figure 2c), 10 families were found exclusively in appendix samples, the Proteobacteria families: Bradyrhizobiaceae, Caulobacteraceae, Comamonadaceae, Methylobacteriaceae, Methylophilaceae, Pseudomonadaceae, and Sphingomonadaceae; two Actinobacteria families: Corynebacteriaceae and Propionibacteriaceae, and the Firmicutes family Christensenellaceae.

Figure 2d shows the profiles of the appendix and fecal samples analyzed at the genus level. In assessing the differentially abundant microbiota reported in this study, we found that *Odoribacter*, *Bilophila*, and *Faecalibacterium* were found in feces and appendix tissue, but *Butyricimonas*, *Lachnobacterium*, and *Megasphaera* were only found within feces (the whole lists of bacteria at the different taxonomic levels found are reported in the Supplementary Material in Table S1).

### 3.3. Odoribacter and Butyricimonas are Related to Insulin Levels

In order to understand the differences found in the biochemical parameters, bacteria that were found differentially abundant between Intact and w/oApp groups were correlated with insulin and triglycerides levels. No statistical differences were found in relation to triglycerides levels. However, insulin levels correlated positively with the levels of *Bilophila* (*r* = 0.403, *p* = 0.012), *Butyricimonas* (*r* = 0.353, *p* = 0.030), *Lachnobacterium* (*r* = 0.322, *p* = 0.048) and *Odoribacter* (*r* = 0.332, *p* = 0.042). If the bacterial levels were assessed according to their study groups, *Butyricimonas* (*r* = 0.610, *p* = 0.006) and *Odoribacter* (*r* = 0.549, *p* = 0.015) were found to be statistically significant within the w/oApp group; however, this significance was not found within the Intact group. The linear regression analysis including these bacterial groups and supporting a correction by age, sex and BMI, showed that only in the case of the w/oApp patients, the levels of *Butyricimonas* and almost significantly *Odoribacter* (*p* = 0.057) could be related to the insulin levels (Table 2).

## 4. Discussion

Making decisions in surgical practice involves careful analysis of the consequences of different options. When making the right decision, short-term concerns as well as long-term consequences should be considered. Incidental prophylactic surgeries are especially relevant because of their preventive nature. In bariatric surgery, the physical examination of the abdomen is generally inaccurate in the period immediately following the surgery, masking appendicitis symptoms [14]. There was a time in which an additional appendectomy was performed in some open bariatric surgeries. This additional appendectomy has been reported without perioperative complications [15]. As no loss of particular and/or vital functions has been found after the appendix removal, the election of an incidental prophylactic appendectomy in open surgeries was ethically justified. However, in the present study, we have found that incidental prophylactic appendectomy could trigger significant changes in gut microbiota diversity, which could induce significant changes in its population in the long-term. This occurrence, usually termed dysbiosis, has been studied in multiple diseases as a risk factor for the deterioration of the health condition [16]. Therefore, appendix removal as a preventive operation could put the patient at risk of losing metabolic homeostasis and developing metabolic diseases.

The Darwinian vision of the appendix function presented this organ as a vestigial organ which evolution will gradually remove in the future. In the last decade, interest in the appendix has emerged and different proposals about its function have been put forward. The “safe house” hypothesis is a very interesting theory focused on the intriguing virtual new organ, the gut microbiota. Thus, the “safe house” hypothesis proposes that the human appendix is a reservoir of healthy microbiota to be replaced in the case of necessity, i.e., profound diarrheas [4]. Thus, appendix microbiota could be linked to the rest of the gut microbiota. The smaller diversity of the gut microbiota of patients who had suffered an incidental prophylactic appendectomy, would suggest that the human appendix could have a role in maintaining the eubiosis in the gut microbiota of the host, supporting the “safe house” hypothesis [4].

The microbiota population of the gastrointestinal tract has an important function within the metabolism of the host [17]. Most of the microbiota members found in the appendix were also found in stool samples, although particular members, mainly from the Proteobacteria phylum, were only found in the appendix tissue. These Proteobacteria families found, in the appendix tissue, the best conditions to live in, and could establish the optimum conditions for the development of other members of the microbiota population. In fact, Proteobacteria have been reported as important actors for maintaining an anaerobic environment of the gut, for the normal microbiome function [18]. Another important point is the fact that some bacteria have been found only in the appendix and within the feces of Intact patients, but not within the feces of the w/oApp group. These facts could be in line with the “safe hypothesis”, as appendix microbiota could be used as the seed for the distal gut microbiota population. However, appendix size has been reported to be too small to affect the migration of its population to the stability of the gut microbiota [19], so further experimental designs would be necessary to decipher this function.

The decrease in the diversity of incidental prophylactic appendectomized patients reported was translated into a worse metabolic scenario for these subjects with an inferior improvement in insulin levels compared to Intact patients. Alpha diversity reflects the complexity of a microbial community, where a higher diversity is usually associated with a healthier microbiome [20].

In particular, the bacteria with different abundances reported in this study are mostly related to metabolic functions: *Odoribacter* is a short chain fatty acid (SCFA) producer, particularly of butyrate. Moreover, it has been related to the levels of blood pressure [21]. *Butyricimonas* is able to reduce the levels of glucose [22] through the production of butyrate. In fact, most of the bacteria reported are related to this SCFA, as *Odoribacter* and *Faecalibacterium*, are also butyrate-producers. Therefore, the prophylactic removal of the appendix could produce a decrease in butyrate production. Butyrate has a central role in promoting and maintaining mucosal homeostasis [23], and a butyrate reduction has been related to inflammatory bowel diseases [24]. On the other hand, *Lachnobacterium*, the genus increased in the w/oApp group, mainly ferments glucose to lactic acid [25]. This could be indicative of a change in the host metabolism and homeostasis. In fact, *Lachnobacterium* has been proposed as a genus predictive of dysbiosis [26]. Finally, *Bilophila* has been recovered from patients with appendicitis [27] and was related to aggravating chronic inflammation in high fat diets [28].

In this study, *Odoribacter*, and especially *Butyricimonas,* have emerged as two important bacteria implicated in insulin regulation. As discussed above, these two genera have been related to positive outcomes in relation to glucose metabolism. Moreover, in previous work, we found a strong negative correlation between *Butyricimonas* and insulin levels [29]. In another interesting study, *Butyricimonas* was related to the beneficial anti-diabetic action of metformin [30]. *Odoribacter* was found to be related to insulin sensitivity in the study by Yamashita et al. using Japanese subjects, but those with a westernized lifestyle exhibited a lower abundance of *Odoribacter* [31]. However, in subjects with their appendix removed, the positive relationship found between the abundance of *Odoribacter* and *Buryricimonas* and insulin levels led us to believe there could be deregulation driven by the low levels found in the w/oApp patients and the Intact patients. Amongst the lower diversity of these patients, this could destabilize the homeostatic symbiosis between the microbial population and the host. In fact, it seems that *Bilophila* co-occurs with *Odoribacter*, and, moreover, this situation may be related to the lower richness and diversity found in w/oApp patients. This finding merits further investigation.

There are some limitations to this observational study. First of all, this pilot study is smaller in scope. Moreover, the lack of stool samples before the appendix removal does not permit us to establish a relationship of causality and confirm our results. However, these new findings could initiate new debates about the introduction of the metabolic consequences led by gut microbiota in the decisions in the practical surgery, and in this manner, other incidental prophylactic appendectomies should be tested.

## 5. Conclusions

In spite of there being no apparent vital function of the human appendix, as well as no particular aggravations of the patients after an incidental prophylactic appendectomy, in this study, we have tested the possibility that the human appendix could serve as a healthy microbiome reservoir. In fact, we have found lower levels of richness and diversity in patients who have suffered from incidental prophylactic appendectomies in the long term, translated into different microbiome profiles related with lower insulin metabolism in these patients. These results suggest that the repercussions on gut microbiota should be introduced into decisions regarding practical surgery.

## Figures and Tables

**Table 1:** Anthropometric and biochemical measurements before bariatric surgery and at the study time.

	Intact Group		w/oApp Group		*p*-Value	
	preSurgery	StudyTime	preSurgery	StudyTime	preSurgery	StudyTime
Age (years)	43.5 ± 9.12	53.65 ± 8.87	38.73 ± 9.25	49.45 ± 9.98		
Gender (M/F)	8/12		5/15			
BMI (kg/m^2^)	52.36 ± 7.33	36.97 ± 7.09 *	54.80 ± 3.81	36.10 ± 6.13 *	0.159	0.682
Waist (cm)	138.26 ± 15.56	116.84 ± 16.97 *	137.00 ± 12.34	111.8 ± 13.50 *	0.850	0.305
Glucose (mg/dL)	119.74 ± 39.86	98.35 ± 37.25 *	113.72 ± 21.84	94.55 ± 24.46 *	0.558	0.705
Cholesterol (mg/dL)	206.21 ± 36.25	151.40 ± 39.30 *	200.06 ± 40.78	140.45 ± 32.91 *	0.618	0.345
HDL-Chol	47.37 ± 12.68	56.60 ± 15.50 *	43.94 ± 10.19	61.10 ± 14.90 *	0.283	0.355
TG (mg/dL)	133.94 ± 57.69	103.75 ± 58.99	143.12 ± 83.61	90.55 ± 37.75 *	0.851	0.405
SBP (mmHg)	141.17 ± 24.86	122.10 ± 14.87 *	141.07 ± 20.20	120.95 ± 14.03 *	0.813	0.803
DBP (mmHg)	81.94 ± 15.72	66.05 ± 7.21 *	84.93 ± 9.51	64.85 ± 8.76 *	0.435	0.639
Insulin (mg/dL)	23.08 ± 12.41	7.93 ± 4.73 *	21.78 ± 12.91	15.70 ± 22.51	0.585	0.149
HOMA-IR	6.38 ± 3.02	2.13 ± 1.81 *	6.21 ± 4.25	4.21 ± 6.67 *	0.730	0.193
CRP (mg/dL)	6.28 ± 7.64	3.44 ± 0.95	5.32 ± 3.64	4.25 ± 2.71	0.656	0.217

Values are presented as means ± SD. BMI, Body Mass Index; HDL-Chol, High Density Lipoprotein cholesterol; TG, Triglycerides; DBP, Diastolic blood pressure; SBP, Systolic blood pressure; HOMA-IR, Homeostatic Model Assessment of Insulin Resistance; CRP, C reactive protein. * Indicates values statistically significant between times within the same treatment group (*p* < 0.05).

**Table 2:** Regression analysis with insulin levels as dependent variable and sex, age, BMI, Bilophila, Butyricimonas, Lachnobacterium and Odoribacter levels as independent variables.

	Insulin Levels					
	Intact (R = 0.717, R^2^ adj = 0.204, *p* = 0.218)			w/oApp (R = 0.818, R^2^ adj = 0.511, *p* = 0.027)		
	ß	*p*-Value	95% CI	ß	*p*-Value	95% CI
**Sex**	−0.370	0.145	−8.508–1.431	0.003	0.987	−22.142–22.470
**Age**	0.340	0.165	−0.090–0.465	−0.163	0.404	−1.335–0.580
**BMI**	0.345	0.144	−0.093–0.563	−0.075	0.692	−1.756–1.208
***Bilophila***	0.465	0.115	−0.671–5.350	−0.169	0.455	−60.468–28.983
***Butyricimonas***	0.101	0.756	−20.423–27.339	0.630	0.011	71.340–448.017
***Lachnobacterium***	0.154	0.614	−24.290–39.289	−0.039	0.855	−27.116–22.873
***Odoribacter***	−0.191	0.634	−14,455–9.189	0.470	0.057	−2.180–132.586

ß: standardized regression coefficient; CI: confidence intervals; BMI, Body Mass Index.

## Supplementary Materials

The following are available online at https://www.mdpi.com/2076-2607/8/4/609/s1, Figure S1: Study design, Table S1: List of bacteria present in feces and appendix tissue.
