# Supplementary material for: Incidental Prophylactic Appendectomy Is Associated with a Profound Microbial Dysbiosis in the Long-Term
Source: Microorganisms. 2020 Apr 23;8(4):609. doi: 10.3390/microorganisms8040609 (PMC7232405; doi:10.3390/microorganisms8040609)

**Phylum Level**

| 2 common elements in "Intact" and "w/oApp": | 6 common elements in "Intact", "w/oApp" and "Appx": |
| --- | --- |
| p__Lentisphaerae | p__Actinobacteria |
| p__Verrucomicrobia | p__Bacteroidetes |
|  | p__Firmicutes |
|  | p__Fusobacteria |
|  | p__Proteobacteria |
|  | p__Synergistetes |


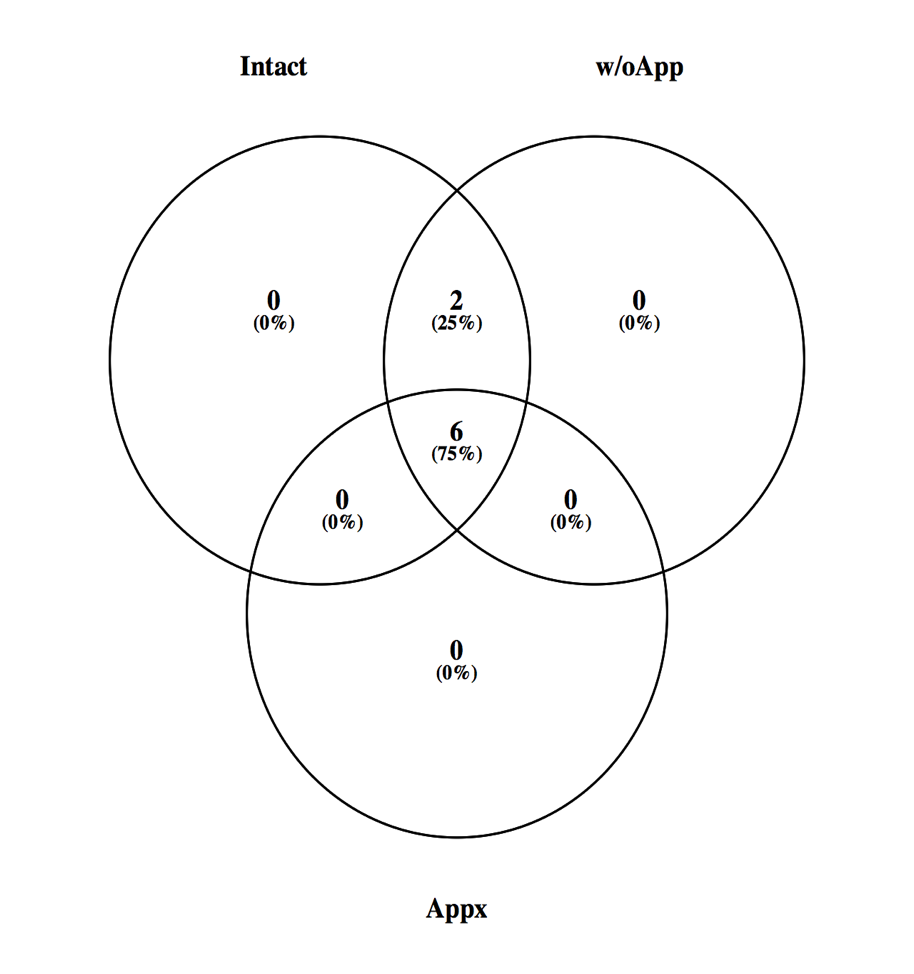


**Family level**

| 2 elements included exclusively in "Intact": | 3 common elements in "Intact" and "w/oApp": | 1 element included exclusively in "w/oApp": | 23 common elements in "Intact", "w/oApp" and "Appx": | 0 common elements in "w/oApp" and "Appx": | 3 common elements in "Intact" and "Appx": | 10 elements included exclusively in "Appx": | |
| --- | --- | --- | --- | --- | --- | --- | --- |
| f__Mogibacteriaceae | f__Carnobacteriaceae | f__Lactobacillaceae | f__Alcaligenaceae |  | f__Barnesiellaceae | f__Bradyrhizobiaceae | |
| f__Peptostreptococcaceae | f__Verrucomicrobiaceae |  | f__Bacteroidaceae |  | f__Clostridiaceae | f__Caulobacteraceae | |
|  | f__Victivallaceae |  | f__Bifidobacteriaceae |  | f__Tissierellaceae | f__Christensenellaceae | |
|  |  |  | f__Coriobacteriaceae |  |  | f__Comamonadaceae | |
|  |  |  | f__Desulfovibrionaceae |  |  | f__Corynebacteriaceae | |
|  |  |  | f__Dethiosulfovibrionaceae |  |  | f__Methylobacteriaceae | |
|  |  |  | f__Enterobacteriaceae |  |  | f__Methylophilaceae | |
|  |  |  | f__Erysipelotrichaceae |  |  | f__Propionibacteriaceae | |
|  |  |  | f__Fusobacteriaceae |  |  | f__Pseudomonadaceae | |
|  |  |  | f__Gemellaceae |  |  | f__Sphingomonadaceae | |
|  |  |  | f__Lachnospiraceae |  |  |  |  |
|  |  |  | f__Odoribacteraceae |  |  |  |  |
|  |  |  | f__Oxalobacteraceae |  |  |  |  |
|  |  |  | f__Paraprevotellaceae |  |  |  |  |
|  |  |  | f__Pasteurellaceae |  |  |  |  |
|  |  |  | f__Porphyromonadaceae |  |  |  |  |
|  |  |  | f__Prevotellaceae |  |  |  |  |
|  |  |  | f__Rikenellaceae |  |  |  |  |
|  |  |  | f__Ruminococcaceae |  |  |  |  |
|  |  |  | f__S24_7 |  |  |  |  |
|  |  |  | f__Streptococcaceae |  |  |  |  |
|  |  |  | f__Succinivibrionaceae |  |  |  |  |
|  |  |  | f__Veillonellaceae |  |  |  |  |
|  |  |  |  |  |  |  |  |
|  |  |  |  |  |  |  |  |


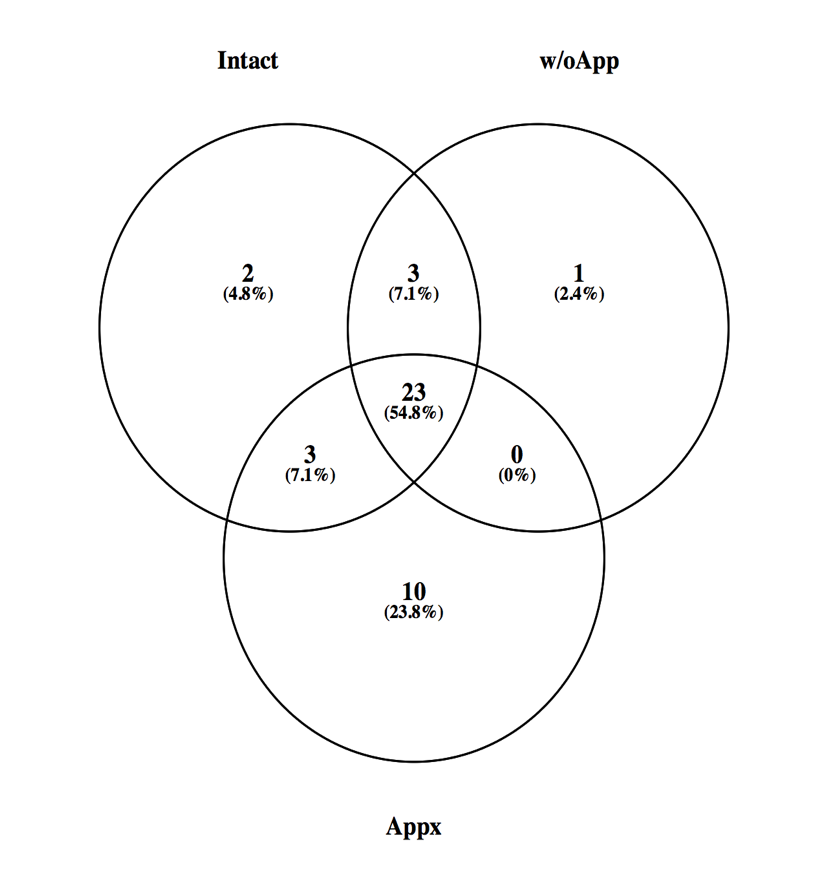


**Genus level**

| 6 elements included exclusively in "Intact": | 2 elements included exclusively in "w/oApp": | 7 common elements in "Intact" and "w/oApp": | 1 common element in "w/oApp" and "Appx": | 2 common elements in "Intact" and "Appx": | | |
| --- | --- | --- | --- | --- | --- | --- |
| g__Alistipes | g__Lactobacillus | g__Akkermansia | g__Succinivibrio | g__Butyricimonas | |  |
| g__Atopobium | g__Megasphaera | g__Granulicatella |  | g__Eubacterium | |  |
| g__Clostridium |  | g__Lachnobacterium |  |  |  |  |
| g__Peptostreptococcus |  | g__Oxalobacter |  |  |  |  |
| g__Sporanaerobacter |  | g__Paraprevotella |  |  |  |  |
| g__Succiniclasticum |  | g__Slackia |  |  |  |  |
|  |  | g__Veillonella |  |  |  |  |
|  |  |  |  |  |  |  |


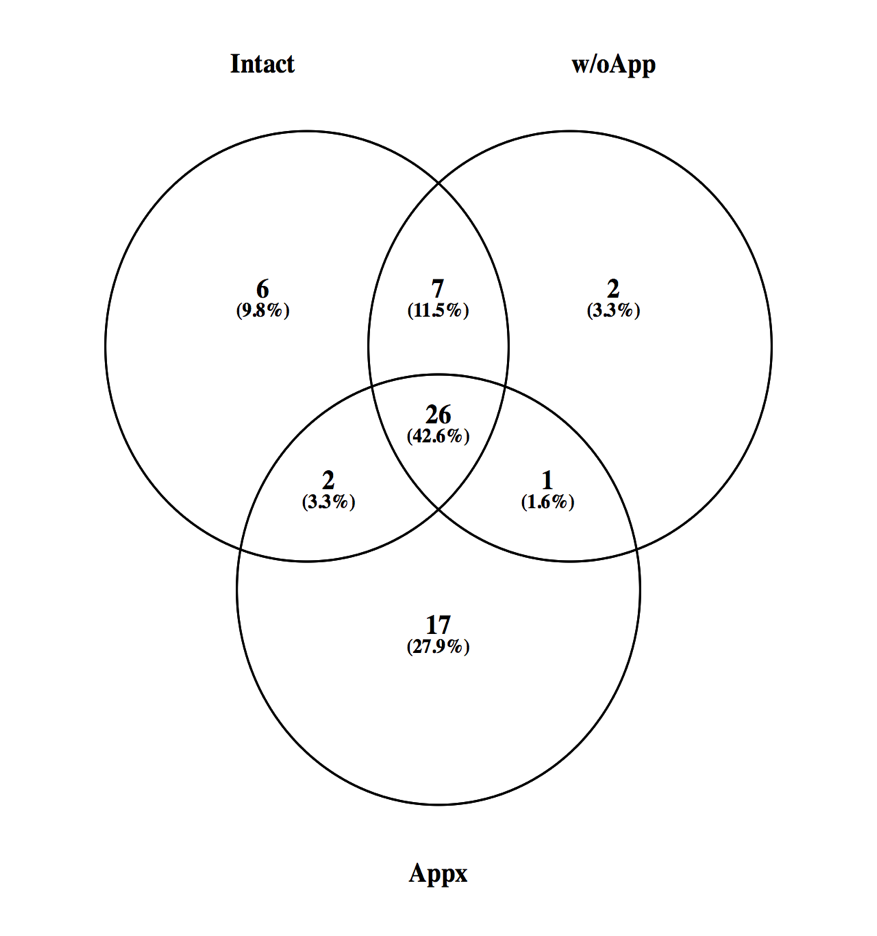

Supplement: Supplementary file 1 [file microorganisms-08-00609-s001.zip › Supplementary material.docx]
