# Supplementary material for: Incidental Prophylactic Appendectomy Is Associated with a Profound Microbial Dysbiosis in the Long-Term
Source: Microorganisms. 2020 Apr 23;8(4):609. doi: 10.3390/microorganisms8040609 (PMC7232405; doi:10.3390/microorganisms8040609)
Supplement: Supplementary file 1 [file microorganisms-08-00609-s001.zip › Figure s1.pdf]

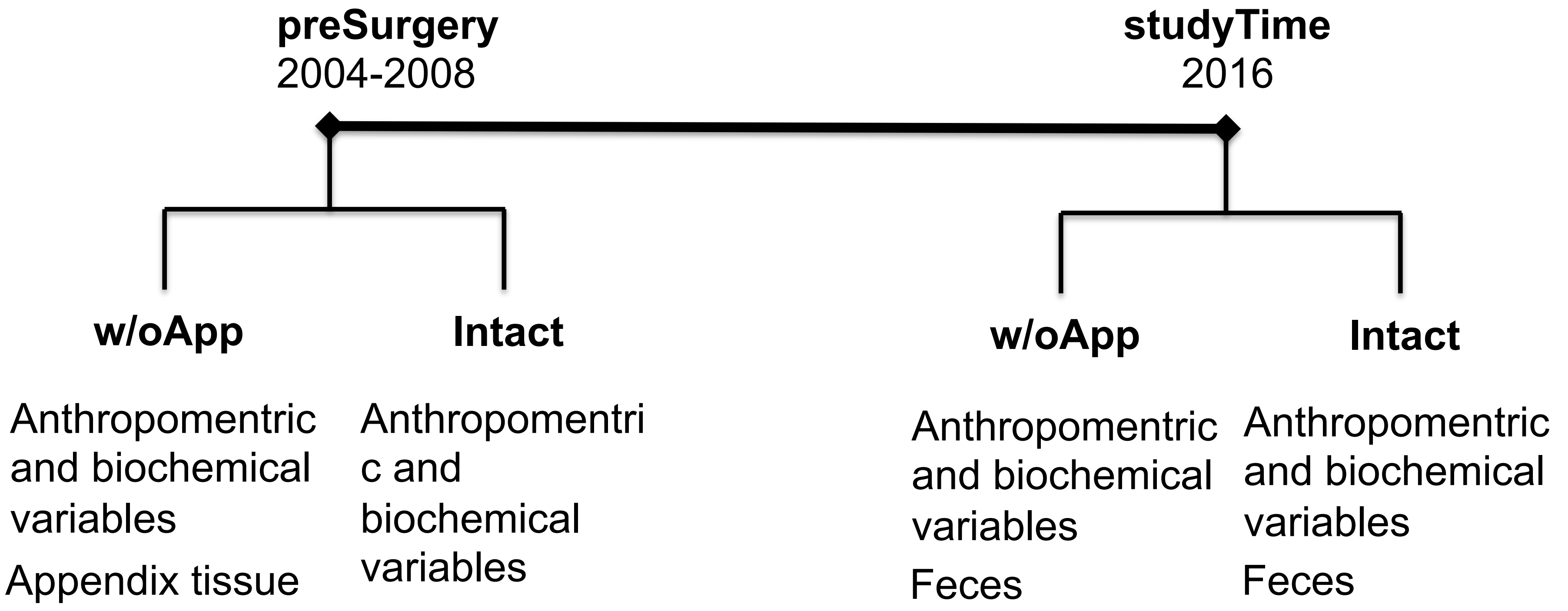
preSurgery
2004-2008
studyTime
2016
w/oApp
Intact
w/oApp
Intact
Anthropomentric and biochemical variables
Appendix tissue
Anthropomentric and biochemical variables
Anthropomentric and biochemical variables
Feces
Anthropomentric and biochemical variables
Feces
